# *S*-1,3-Benzothia­zol-2-yl (2*Z*)-2-(2-amino-1,3-thia­zol-4-yl)-2-(methoxy­imino)­ethane­thio­ate

**DOI:** 10.1107/S1600536809025616

**Published:** 2009-07-11

**Authors:** Shahzad Sharif, Islam Ullah Khan, Muhammad Nadeem Arshad, Tahir Ali Sheikh, Muhammad Zahid Qureshi

**Affiliations:** a Materials Chemistry Laboratory, Department of Chemistry, GC University, Lahore, Pakistan

## Abstract

The title compound, C_13_H_10_N_4_O_2_S_3_, is an acyl­ating agent which belongs to the thia­zole class of organic compounds. The dihedral angle between the benzene and thiazole rings, which are fused to each other, is 1.2 (2)°  so the overall benzothiazole system is almost planar. Inter­molecular N—H⋯N inter­actions and weak C—H⋯O inter­actions between symmetry-related mol­ecules stabilize the crystal structure, forming three different ring motifs [*R*_2_^2^(8), *R*_2_^2^(10) and *R*_2_^2^(16)] in three dimensions.

## Related literature

For background literature, see: Khanna *et al.* (1999 ▶). For related structures, see: Radha (1985 ▶); Laurent & Durant (1981 ▶). For graph set notation, see: Bernstein *et al.* (1995 ▶).
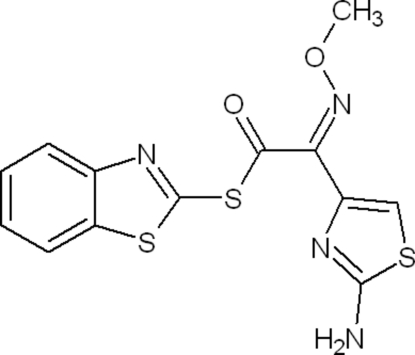


## Experimental

### 

#### Crystal data

C_13_H_10_N_4_O_2_S_3_
*M* *_r_* = 350.43
Monoclinic, 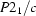

*a* = 13.9725 (9) Å
*b* = 5.0156 (3) Å
*c* = 21.7664 (14) Å
β = 90.001 (3)°
*V* = 1525.40 (17) Å^3^
*Z* = 4
Mo *K*α radiation
μ = 0.50 mm^−1^
*T* = 296 K
0.31 × 0.11 × 0.07 mm

#### Data collection

Bruker Kappa APEXII CCD diffractometer
Absorption correction: multi-scan (*SADABS*; Bruker, 2007 ▶) *T*_min_ = 0.941, *T*_max_ = 0.972
16828 measured reflections
3829 independent reflections
2096 reflections with *I* > 2σ(*I*)
*R*_int_ = 0.054

#### Refinement

*R*[*F* ^2^ > 2σ(*F* ^2^)] = 0.046
*wR*(*F* ^2^) = 0.145
*S* = 1.03
3829 reflections
200 parameters
H-atom parameters constrained
Δρ_max_ = 0.35 e Å^−3^
Δρ_min_ = −0.34 e Å^−3^

### 

Data collection: *APEX2* (Bruker, 2007 ▶); cell refinement: *SAINT* (Bruker, 2007 ▶); data reduction: *SAINT*; program(s) used to solve structure: *SHELXS97* (Sheldrick, 2008 ▶); program(s) used to refine structure: *SHELXL97* (Sheldrick, 2008 ▶); molecular graphics: *ORTEP-3 for Windows* (Farrugia, 1997 ▶) and *PLATON* (Spek, 2009 ▶); software used to prepare material for publication: *WinGX* (Farrugia, 1999 ▶) and *PLATON*.

## Supplementary Material

Crystal structure: contains datablocks I, global. DOI: 10.1107/S1600536809025616/pv2172sup1.cif

Structure factors: contains datablocks I. DOI: 10.1107/S1600536809025616/pv2172Isup2.hkl

Additional supplementary materials:  crystallographic information; 3D view; checkCIF report

## Figures and Tables

**Table 1:** Hydrogen-bond geometry (Å, °)

*D*—H⋯*A*	*D*—H	H⋯*A*	*D*⋯*A*	*D*—H⋯*A*
N3—H3*A*⋯N1^i^	0.86	2.29	3.106 (3)	158
N3—H3*B*⋯N4^i^	0.86	2.16	2.997 (3)	165
C12—H7⋯O1^ii^	0.93	2.51	3.198 (3)	131

Symmetry codes: (i) 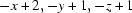; (ii) 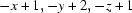.

**Fig. 1.**
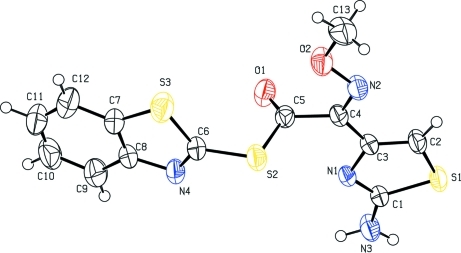
The structure of (I) with displacement ellipsoids for the non-hydrogen atoms drawn at the 50% probability level.

**Fig. 2.**
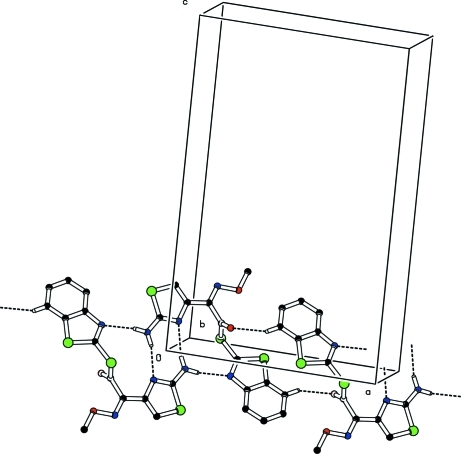
Unit cell packing for (I) showing hydrogen bonds drawn as dashed lines.

## supplementary crystallographic information

### Comment

The title compound, (I), is a standard acylating agent for the synthesis of different cephalosporins derivatives (Khanna *et al.*, 1999).

The title compound (Fig. 1) is composed of two components, a benzothiazole and an acyl group having 2-amino thiazole moiety. The N3 atoms of 2-aminothiazole moieties of molecules of (I) lying about inversion centers form intermolecular hydrogen bonds of the type N–H···N with N1 and N4 atoms resulting in dimers and form 8 and 10-membered rings which could be expressed in graph set notation as R_2_^2^(8) and R_2_^2^(10) motifs (Bernstein *et al.*, 1995). In addition, there is a rather weak C–H···O type interaction, resulting in an R_2_^2^(16) motif about inversion centers. The hydrogen bonding geometry is presented in Table 1 and Fig. 2. The crystal structures of compounds related to (I) have been reported (Radha, 1985; Laurent & Durant, 1981) .

### Experimental

The compound was dissolved in methanol and ethyl acetate mixture (20:80 *v*/*v* %). The light yellow prismatic crystals were obtained after two days.

### Refinement

The H-atoms were refined geometrically and treated as riding atoms with C—H distances, 0.93 and 0.96 Å for aryl and methyl groups, respectively, and N–H = 0.86 Å with *U*_iso_(H) = 1.2 times aromatic C and N atoms and *U*_iso_(H) = 1.5 times methyl C atoms.

### Crystal data

**Table:**

C_13_H_10_N_4_O_2_S_3_	*F*(000) = 720
*M**_r_* = 350.43	*D*_x_ = 1.526 Mg m^−^^3^
Monoclinic, *P*2_1_/*c*	Mo *K*α radiation, λ = 0.71073 Å
Hall symbol: -P 2ybc	Cell parameters from 2744 reflections
*a* = 13.9725 (9) Å	θ = 2.4–22.8°
*b* = 5.0156 (3) Å	µ = 0.50 mm^−^^1^
*c* = 21.7664 (14) Å	*T* = 296 K
β = 90.001 (3)°	Prism, light yellow
*V* = 1525.40 (17) Å^3^	0.31 × 0.11 × 0.07 mm
*Z* = 4	

### Data collection

**Table:**

Bruker Kappa APEXII CCD diffractometer	3829 independent reflections
Radiation source: fine-focus sealed tube	2096 reflections with *I* > 2σ(*I*)
graphite	*R*_int_ = 0.054
φ and ω scans	θ_max_ = 28.4°, θ_min_ = 1.5°
Absorption correction: multi-scan (*SADABS*; Bruker, 2007)	*h* = −18→18
*T*_min_ = 0.941, *T*_max_ = 0.972	*k* = −6→6
16828 measured reflections	*l* = −29→28

### Refinement

**Table:**

Refinement on *F*^2^	Primary atom site location: structure-invariant direct methods
Least-squares matrix: full	Secondary atom site location: difference Fourier map
*R*[*F*^2^ > 2σ(*F*^2^)] = 0.046	Hydrogen site location: inferred from neighbouring sites
*wR*(*F*^2^) = 0.145	H-atom parameters constrained
*S* = 1.03	*w* = 1/[σ^2^(*F*_o_^2^) + (0.0695*P*)^2^] where *P* = (*F*_o_^2^ + 2*F*_c_^2^)/3
3829 reflections	(Δ/σ)_max_ = 0.001
200 parameters	Δρ_max_ = 0.35 e Å^−^^3^
0 restraints	Δρ_min_ = −0.34 e Å^−^^3^

### Special details

**Table:**

Geometry. All e.s.d.'s (except the e.s.d. in the dihedral angle between two l.s. planes) are estimated using the full covariance matrix. The cell e.s.d.'s are taken into account individually in the estimation of e.s.d.'s in distances, angles and torsion angles; correlations between e.s.d.'s in cell parameters are only used when they are defined by crystal symmetry. An approximate (isotropic) treatment of cell e.s.d.'s is used for estimating e.s.d.'s involving l.s. planes.
Refinement. Refinement of *F*^2^ against ALL reflections. The weighted *R*-factor *wR* and goodness of fit *S* are based on *F*^2^, conventional *R*-factors *R* are based on *F*, with *F* set to zero for negative *F*^2^. The threshold expression of *F*^2^ > σ(*F*^2^) is used only for calculating *R*-factors(gt) *etc*. and is not relevant to the choice of reflections for refinement. *R*-factors based on *F*^2^ are statistically about twice as large as those based on *F*, and *R*- factors based on ALL data will be even larger.

### Fractional atomic coordinates and isotropic or equivalent isotropic displacement parameters (Å^2^)

**Table:**

	*x*	*y*	*z*	*U*_iso_*/*U*_eq_	
S1	1.09909 (5)	0.53325 (15)	0.33403 (3)	0.0473 (2)	
S2	0.82219 (5)	1.20705 (16)	0.47275 (4)	0.0481 (2)	
S3	0.62042 (6)	1.30442 (19)	0.51641 (4)	0.0658 (3)	
O1	0.73807 (14)	0.7981 (4)	0.41758 (10)	0.0515 (6)	
O2	0.76884 (15)	1.2729 (4)	0.32595 (10)	0.0599 (6)	
N1	0.97196 (15)	0.6544 (4)	0.41510 (10)	0.0342 (5)	
N2	0.85575 (16)	1.1371 (5)	0.31772 (11)	0.0457 (6)	
N3	1.07870 (17)	0.3179 (5)	0.44609 (11)	0.0508 (7)	
H3A	1.0515	0.3001	0.4813	0.061*	
H3B	1.1273	0.2209	0.4368	0.061*	
N4	0.73303 (16)	0.9626 (5)	0.56704 (11)	0.0468 (6)	
C1	1.04521 (18)	0.4975 (5)	0.40561 (12)	0.0352 (6)	
C2	1.0159 (2)	0.7693 (5)	0.31617 (13)	0.0428 (7)	
H1	1.0129	0.8598	0.2789	0.051*	
C3	0.95521 (18)	0.8065 (5)	0.36329 (12)	0.0340 (6)	
C4	0.87134 (19)	0.9811 (5)	0.36324 (12)	0.0370 (6)	
C5	0.80041 (19)	0.9594 (5)	0.41574 (13)	0.0379 (7)	
C6	0.72758 (19)	1.1345 (6)	0.52336 (14)	0.0427 (7)	
C7	0.5768 (2)	1.1236 (6)	0.57791 (14)	0.0516 (8)	
C8	0.6472 (2)	0.9518 (6)	0.59907 (14)	0.0451 (7)	
C9	0.6272 (2)	0.7821 (7)	0.64769 (16)	0.0641 (10)	
H10	0.6733	0.6639	0.6622	0.077*	
C10	0.5376 (3)	0.7925 (8)	0.67398 (17)	0.0729 (11)	
H9	0.5232	0.6798	0.7066	0.088*	
C11	0.4697 (3)	0.9652 (8)	0.65302 (19)	0.0755 (11)	
H8	0.4099	0.9676	0.6719	0.091*	
C12	0.4867 (2)	1.1338 (8)	0.60535 (19)	0.0762 (11)	
H7	0.4400	1.2516	0.5916	0.091*	
C13	0.7520 (3)	1.4460 (6)	0.27606 (16)	0.0641 (10)	
H11A	0.8078	1.5537	0.2691	0.096*	
H11B	0.6985	1.5590	0.2852	0.096*	
H11C	0.7384	1.3431	0.2399	0.096*	

### Atomic displacement parameters (Å^2^)

**Table:**

	*U*^11^	*U*^22^	*U*^33^	*U*^12^	*U*^13^	*U*^23^
S1	0.0469 (4)	0.0640 (5)	0.0309 (4)	0.0148 (4)	0.0129 (3)	0.0034 (4)
S2	0.0494 (5)	0.0563 (5)	0.0387 (5)	−0.0072 (4)	0.0129 (4)	−0.0009 (4)
S3	0.0557 (5)	0.0812 (6)	0.0605 (6)	0.0223 (4)	0.0143 (4)	0.0168 (5)
O1	0.0441 (12)	0.0557 (12)	0.0547 (15)	−0.0042 (10)	0.0139 (10)	−0.0033 (10)
O2	0.0565 (13)	0.0793 (15)	0.0439 (14)	0.0303 (11)	0.0118 (11)	0.0226 (11)
N1	0.0349 (12)	0.0442 (13)	0.0236 (13)	0.0022 (10)	0.0041 (10)	0.0040 (10)
N2	0.0469 (14)	0.0550 (14)	0.0353 (15)	0.0137 (12)	0.0067 (11)	0.0056 (12)
N3	0.0567 (16)	0.0613 (15)	0.0342 (15)	0.0246 (13)	0.0114 (12)	0.0107 (13)
N4	0.0419 (14)	0.0621 (15)	0.0363 (15)	0.0042 (12)	0.0108 (11)	0.0046 (13)
C1	0.0354 (14)	0.0429 (15)	0.0274 (15)	−0.0001 (12)	0.0043 (12)	−0.0033 (13)
C2	0.0465 (16)	0.0536 (17)	0.0283 (16)	0.0077 (13)	0.0091 (13)	0.0076 (13)
C3	0.0369 (14)	0.0399 (14)	0.0251 (15)	−0.0006 (12)	0.0031 (12)	0.0006 (12)
C4	0.0399 (15)	0.0444 (16)	0.0266 (16)	0.0013 (12)	0.0046 (12)	0.0021 (13)
C5	0.0359 (14)	0.0439 (16)	0.0339 (17)	0.0095 (13)	0.0040 (12)	0.0060 (13)
C6	0.0391 (15)	0.0539 (17)	0.0351 (17)	0.0019 (13)	0.0079 (13)	−0.0053 (14)
C7	0.0427 (16)	0.069 (2)	0.043 (2)	−0.0017 (15)	0.0105 (14)	−0.0055 (16)
C8	0.0426 (16)	0.0606 (19)	0.0320 (17)	−0.0018 (14)	0.0081 (13)	−0.0045 (15)
C9	0.060 (2)	0.084 (2)	0.049 (2)	−0.0032 (18)	0.0113 (17)	0.0124 (19)
C10	0.077 (3)	0.095 (3)	0.046 (2)	−0.021 (2)	0.020 (2)	0.006 (2)
C11	0.052 (2)	0.109 (3)	0.066 (3)	−0.010 (2)	0.0231 (19)	−0.011 (2)
C12	0.047 (2)	0.109 (3)	0.072 (3)	0.013 (2)	0.0178 (19)	0.001 (2)
C13	0.075 (2)	0.066 (2)	0.052 (2)	0.0228 (18)	−0.0030 (18)	0.0160 (18)

### Geometric parameters (Å, °)

**Table:**

S1—C2	1.704 (3)	C2—C3	1.344 (4)
S1—C1	1.740 (3)	C2—H1	0.9300
S2—C6	1.759 (3)	C3—C4	1.463 (4)
S2—C5	1.782 (3)	C4—C5	1.517 (3)
S3—C7	1.728 (3)	C7—C8	1.387 (4)
S3—C6	1.729 (3)	C7—C12	1.394 (4)
O1—C5	1.190 (3)	C8—C9	1.387 (4)
O2—N2	1.404 (3)	C9—C10	1.378 (5)
O2—C13	1.410 (4)	C9—H10	0.9300
N1—C1	1.308 (3)	C10—C11	1.364 (5)
N1—C3	1.382 (3)	C10—H9	0.9300
N2—C4	1.281 (3)	C11—C12	1.360 (5)
N3—C1	1.344 (3)	C11—H8	0.9300
N3—H3A	0.8600	C12—H7	0.9300
N3—H3B	0.8600	C13—H11A	0.9600
N4—C6	1.286 (4)	C13—H11B	0.9600
N4—C8	1.388 (3)	C13—H11C	0.9600
			
C2—S1—C1	88.90 (13)	N4—C6—S2	123.9 (2)
C6—S2—C5	99.42 (13)	S3—C6—S2	119.61 (18)
C7—S3—C6	88.79 (15)	C8—C7—C12	121.4 (3)
N2—O2—C13	110.1 (2)	C8—C7—S3	109.5 (2)
C1—N1—C3	109.6 (2)	C12—C7—S3	129.1 (3)
C4—N2—O2	110.2 (2)	C9—C8—C7	119.5 (3)
C1—N3—H3A	120.0	C9—C8—N4	125.6 (3)
C1—N3—H3B	120.0	C7—C8—N4	115.0 (3)
H3A—N3—H3B	120.0	C10—C9—C8	118.5 (3)
C6—N4—C8	110.3 (2)	C10—C9—H10	120.8
N1—C1—N3	124.9 (2)	C8—C9—H10	120.8
N1—C1—S1	114.7 (2)	C11—C10—C9	121.2 (4)
N3—C1—S1	120.37 (19)	C11—C10—H9	119.4
C3—C2—S1	110.7 (2)	C9—C10—H9	119.4
C3—C2—H1	124.7	C12—C11—C10	121.9 (3)
S1—C2—H1	124.7	C12—C11—H8	119.1
C2—C3—N1	116.1 (2)	C10—C11—H8	119.1
C2—C3—C4	126.0 (2)	C11—C12—C7	117.5 (4)
N1—C3—C4	117.8 (2)	C11—C12—H7	121.2
N2—C4—C3	120.2 (2)	C7—C12—H7	121.2
N2—C4—C5	121.0 (2)	O2—C13—H11A	109.5
C3—C4—C5	118.7 (2)	O2—C13—H11B	109.5
O1—C5—C4	123.6 (3)	H11A—C13—H11B	109.5
O1—C5—S2	125.2 (2)	O2—C13—H11C	109.5
C4—C5—S2	111.29 (19)	H11A—C13—H11C	109.5
N4—C6—S3	116.5 (2)	H11B—C13—H11C	109.5
			
C13—O2—N2—C4	179.6 (3)	C8—N4—C6—S3	0.8 (3)
C3—N1—C1—N3	−177.9 (3)	C8—N4—C6—S2	179.6 (2)
C3—N1—C1—S1	1.6 (3)	C7—S3—C6—N4	−0.9 (3)
C2—S1—C1—N1	−1.1 (2)	C7—S3—C6—S2	−179.7 (2)
C2—S1—C1—N3	178.4 (2)	C5—S2—C6—N4	87.6 (3)
C1—S1—C2—C3	0.3 (2)	C5—S2—C6—S3	−93.60 (19)
S1—C2—C3—N1	0.6 (3)	C6—S3—C7—C8	0.7 (2)
S1—C2—C3—C4	−176.0 (2)	C6—S3—C7—C12	−179.4 (4)
C1—N1—C3—C2	−1.4 (3)	C12—C7—C8—C9	1.4 (5)
C1—N1—C3—C4	175.5 (2)	S3—C7—C8—C9	−178.6 (3)
O2—N2—C4—C3	176.5 (2)	C12—C7—C8—N4	179.6 (3)
O2—N2—C4—C5	0.7 (4)	S3—C7—C8—N4	−0.4 (3)
C2—C3—C4—N2	−7.4 (4)	C6—N4—C8—C9	177.9 (3)
N1—C3—C4—N2	176.1 (2)	C6—N4—C8—C7	−0.3 (4)
C2—C3—C4—C5	168.5 (3)	C7—C8—C9—C10	−0.8 (5)
N1—C3—C4—C5	−8.1 (4)	N4—C8—C9—C10	−178.8 (3)
N2—C4—C5—O1	92.6 (3)	C8—C9—C10—C11	0.0 (6)
C3—C4—C5—O1	−83.3 (3)	C9—C10—C11—C12	0.2 (6)
N2—C4—C5—S2	−86.7 (3)	C10—C11—C12—C7	0.4 (6)
C3—C4—C5—S2	97.4 (2)	C8—C7—C12—C11	−1.2 (6)
C6—S2—C5—O1	−1.4 (3)	S3—C7—C12—C11	178.8 (3)
C6—S2—C5—C4	177.9 (2)		

### Hydrogen-bond geometry (Å, °)

**Table:**

*D*—H···*A*	*D*—H	H···*A*	*D*···*A*	*D*—H···*A*
N3—H3A···N1^i^	0.86	2.29	3.106 (3)	158
N3—H3B···N4^i^	0.86	2.16	2.997 (3)	165
C12—H7···O1^ii^	0.93	2.51	3.198 (3)	131

Symmetry codes: (i) −*x*+2, −*y*+1, −*z*+1; (ii) −*x*+1, −*y*+2, −*z*+1.
